# Antioxidant and Cytoprotective Effects of Chilean Macroalgae Against Oxidative Stress-Induced Damage in Gastric Epithelial Cells

**DOI:** 10.3390/nu18121878

**Published:** 2026-06-10

**Authors:** Samantha Acevedo-Correa, Paola A. Haeger, Francisco Álvarez, Michael Araya, Fadia Tala, Erwin de la Fuente-Ortega

**Affiliations:** 1 Laboratorio de Estrés Celular y Enfermedades Crónicas no Transmisibles, Universidad Católica del Norte, Coquimbo 1781421, Chile; 2 Núcleo de Investigación en Prevención y Tratamiento de Enfermedades Crónicas no Transmisibles (NiPTEC), Universidad Católica del Norte, Coquimbo 1781421, Chile; 3 Millennium Nucleus of Neuroepigenetics and Plasticity (EpiNeuro), Santiago 8320000, Chile; 4 Centro de Investigación y Desarrollo Tecnológico en Algas y Otros Recursos Biológicos (CIDTA), Facultad de Ciencias del Mar, Universidad Católica del Norte, Coquimbo 1781421, Chile; 5 Departamento de Biología Marina, Facultad de Ciencias del Mar, Universidad Católica del Norte, Coquimbo 1781421, Chile; 6 Instituto Milenio en Socio-Ecología Costera (SECOS), Santiago 7550000, Chile

**Keywords:** macroalgae extracts, oxidative stress, antioxidants, gastric epithelial cells, polyphenols, gastric diseases

## Abstract

**Background/Objectives**: Oxidative stress is a key pathogenic factor in gastric diseases (GDs). Nutraceuticals with antioxidant activity derived from macroalgae represent promising preventive strategies. However, Chilean macroalgae remains poorly explored in the context of GDs, particularly associated with oxidative stress. This study evaluated the antioxidant and cytoprotective properties of crude aqueous and ethanolic extracts from green, brown, and red macroalgae collected along the north–central coast of Chile. **Methods**: Crude extracts were prepared from green, brown, and red macroalgae and evaluated for antioxidant activity via ABTS, DPPH, and FRAP assays. Using hydrogen peroxide-induced oxidative stress in GES-1 gastric epithelial cells, we assessed cell viability (MTS assay), intracellular reactive oxygen species (ROS) levels (time-lapse confocal microscopy), and apoptosis (active caspase-3 detection). **Results**: All extracts exhibited antioxidant activity; the red macroalgae *Gracilaria chilensis* displayed the highest flavonoid content (up to 2.24 mg QE/g dw). Notably, extracts from *G. chilensis*, *S. gaudichaudii*, and *M. canaliculata* preserved GES-1 cell viability under hydrogen peroxide-induced stress, outperforming green and brown species, demonstrating the superior cytoprotective capacity of red macroalgae compared to other groups. Furthermore, *G. chilensis* extracts significantly reduced intracellular ROS levels and attenuated ROS-induced apoptosis. **Conclusions**: Red macroalgae extracts, particularly *G. chilensis*, exhibit strong antioxidant and cytoprotective effects. Our findings demonstrate that these species outperform green and brown macroalgae, addressing a gap in knowledge regarding Chilean marine resources. These results support their potential development as nutraceuticals for the prevention of oxidative stress-related gastric diseases and highlight red macroalgae as a valuable source of bioactive compounds for diet-based preventive strategies.

## 1. Introduction

Gastric diseases (GDs) are a significant public health problem that requires new treatment approaches. GDs represent a spectrum of disorders, including chronic gastritis, gastric ulcers, functional dyspepsia, and gastric cancer (GC), which are highly prevalent [1]. Currently, GC ranks as the fourth leading cause of cancer death and the fifth most common cancer with over one million new cases annually [2,3,4,5]. Although conventional pharmacological treatments for gastric disorders, such as H_2_ receptor antagonists and proton pump inhibitors, are widely used, their long-term administration can lead to adverse effects, emphasizing the need for safer and natural alternatives [6]. Historically, plant-derived natural products have demonstrated therapeutic efficacy and safety, representing a promising basis for the development of novel nutritional supplements and functional foods [7]. Nutraceuticals (foods or food-derived compounds with health-promoting and medicinal properties) have gained increasing attention for their ability to maintain gastric homeostasis and prevent disease onset [8]. In GDs, oxidative stress is the central pathological mechanism and a potential therapeutic target [9,10,11]. Oxidative stress arises from a redox imbalance between the increased production of reactive oxygen species (ROS) and/or decreased antioxidant defenses [9,12,13]. In low levels, ROS—superoxide, hydroxyl radical, and hydrogen peroxide (H_2_O_2_)—play key roles in cell signaling, immune defense, tissue repair, and the regulation of apoptosis [10,11,12,14]. However, in GDs, factors such as cigarette smoking, chronic alcohol consumption, nonsteroidal anti-inflammatory drug (NSAID), and *Helicobacter pylori* (*H. pylori*) infection can trigger excessive ROS production and oxidative stress [11,12,15]. Oxidative stress can induce apoptosis in gastric epithelial cells, thereby compromising mucosal integrity and barrier function [11,16,17,18]. In addition, oxidative stress increases mucosal permeability, driving the pathogenesis and progression of chronic gastrointestinal disorders, including *H. pylori*-associated gastritis, reflux esophagitis, and gastric carcinogenesis [18,19,20,21,22]. Therefore, oxidative stress represents a therapeutic target for GDs, with exogenous antioxidants from natural products being an effective and economical approach to prevent these diseases [8,12].

Several studies worldwide have reported that macroalga extracts exhibit gastroprotective effects. Extracts from brown and red macroalgae collected in the Americas and Asia have been shown to enhance the gastric mucosal defense and protect against mucosal injury in both cellular and murine models [23,24,25,26,27]. For example, an extract from red macroalgae *Gracilaria changii* (Malaysia) demonstrated strong gastroprotective and anti-ulcerogenic effects in rats, significantly reducing lesion size in ethanol-induced gastric injury [23]. Extracts from *Gracilaria caudata* collected along the Brazilian coast, enriched with a sulfated-polysaccharide (PLS), have shown protective effects against ethanol-induced gastric damage in mice [25,28]. Similarly, an extract from the brown macroalgae *Sargassum polycystum* (India) reversed ethanol/HCl-induced gastric damage in rat models [24]. In addition, an extract from Spanish macroalgae *Gongolaria baccata* has shown protective effects in Caco-2 cells [29]. However, the bioactivity of macroalgae is often site-dependent, as geographic location, seasonality, and local environmental conditions can significantly influence their chemical composition and biological activity, thereby affecting reproducibility and standardization [30,31,32,33]. Although Chile possesses extensive macroalgal resources along its coastline [34], comparative evidence across macroalgal groups under standardized conditions in gastric epithelial models of oxidative stress remains scarce, and their nutraceutical potential is still poorly understood.

In this study, we prepared crude ethanolic and aqueous extracts from green (*Ulva* sp.), brown (*Lessonia spicata*, *Macrocystis pyrifera*), and red (*Gracilaria chilensis*, *Sarcodiotheca gaudichaudii*, and *Mazzaella canaliculate*) Chilean macroalgae to perform a comparative analysis of their antioxidant capacity both in vitro and over a human gastric epithelial cell line (GES-1) exposed to oxidative stress. This work represents a preliminary exploratory study in this area, in which we found that, while all the macroalgal extracts possessing antioxidant capacity, the ethanolic fractions—particularly from *G. chilensis*—showed the most pronounced protective effects on GES-1 cells under peroxide-induced oxidative stress, enhancing cell viability, reducing intracellular ROS, and apoptosis. These findings highlight the superior cytoprotective potential of red macrolagae and support their development as nutraceuticals for the prevention of oxidative stress-related gastric diseases.

## 2. Materials and Methods

### 2.1. Materials

Hydrogen peroxide (#107210) and Cisplatin (#15663, Merck Millipore) were obtained from Merck Millipore (Darmstadt, Germany). Cisplatin was solubilized in PBS 1X (140 mM NaCl). MTS assay was purchased from Promega (Madison, WI, USA). Anti-Cleaved Caspase 3 (#D175) was purchased from Cell Signaling Technologies (Danvers, MA, USA). The Alexa Fluor 488 anti-rabbit secondary antibody (#A11008) and DCFH-DA (2′,7′-dichlorofluorescein diacetate) (#D6883) were purchased from Thermo Fisher Scientific (Eugene, OR, USA).

To obtain the macroalgae extracts, an Elma ultrasonic bath (model D-78224 Singen), a Daihan Scientific Wisemix orbital shaker, a Büchi rotary evaporator (model R-210), and an Ilshin freeze dryer (model FD 8508) were used. Ethanolic extracts were performed with Ethanolic pa. and with pure water. Trolox (#53188-07-1), gallic acid (#149-91-7), and quercetin (#6151-25-3) were used as antioxidant, phenol, and flavonoid standards provided by Sigma-Aldrich. All standard solutions were prepared using distilled and deionized water. Absorbance measurements for each method were performed on a BIOTEK Synergy LX multiplate reader (Biotek Instruments, Winooski, VT, USA).

### 2.2. Macroalgae Collection and Crude Extracts Preparation

Green, brown, and red macroalgae were collected from different sites in central-northern Chile (Table 1). The samples were transported to the laboratory in thermal containers and subsequently washed with distilled water to remove salts and adhering particles. The macroalgae were dried at 50 °C (<20% moisture) and then pulverized with a Micro Ball Mill GT300 (Powteq, Beijing, China) to 100–200 µm powder granulation for analysis and extract generation.

As previously mentioned, ethanolic extracts of macroalgae were obtained from dried and ground biomass. Ten grams of dried sample were weighed and extracted with 70% (*v*/*v*) aqueous ethanol at a solid/solvent ratio of 1:10 (g/mL) using ultrasound-assisted extraction in a Singer D-78224 ultrasonic bath (Elma) for 15 min, followed by shaking on a Wisemix orbital shaker (Daihan Scientific) at 130 rpm for 24 h, according to methodologies described for the extraction of phenolic compounds from macroalgae with aqueous ethanol. The extracts were separated by filtration, and the solvent was removed under reduced pressure in a rotavapor (R-210, Büchi) to dryness. The resulting solid was subjected to solubility separation to obtain a water-soluble fraction and an absolute ethanol-soluble fraction, which were lyophilized and stored at −20 °C.

### 2.3. Total Phenols and Flavonoids Quantification

Phenolic compounds were extracted from 10–100 mg of dried sample using 1 mL of 70% (*v*/*v*) ethanol. Quantification was carried out using the Folin–Ciocalteu method, with gallic acid (20–100 mg/L) used as the standard [35]. Briefly, 0.5 mL of extract or standard solution was mixed with 2.5 mL of Folin–Ciocalteu reagent (Merck) previously diluted 1:10 (*v*/*v*). After 5 min of reaction, 2 mL of 7.5% (*w*/*v*) Na_2_CO_3_ was added. The mixture was incubated in the dark for 30 min at room temperature, and absorbance was measured at 765 nm. Results were expressed as mg gallic acid equivalents (GAE) per gram of dry weight (DW).

Flavonoids were extracted from 10–100 mg of dried sample using 1 mL of 80% (*v*/*v*) ethanol. Quantification was performed using the aluminum chloride colorimetric method, with quercetin (Sigma-Aldrich) used as the standard [36]. Briefly, 0.5 mL of extract or standard solution was mixed with 2 mL of distilled water in a 5 mL volumetric flask. Subsequently, 0.15 mL of 5% NaNO_2_ was added (t = 0 min), followed by 0.15 mL of 10% AlCl_3_ (t = 5 min), and 1 mL of 1 M NaOH (t = 6 min). The final volume was adjusted with distilled water and the mixture was thoroughly homogenized. Absorbance was measured at 510 nm. Results were expressed as mg quercetin equivalents (QE) per gram of dry weight (DW).

### 2.4. Antioxidant Capacity

The antioxidant capacity of the six macroalgae was evaluated using ABTS, DPPH, and FRAP assays [37,38]. Additionally, the antioxidant capacity of the raw macroalgae was measured directly without any prior extraction process. For this assessment, the six seaweed samples were dried and pulverized, and the results were expressed as Trolox equivalents (TEAC, µmol/g of dry algae) using a calibration curve constructed with Trolox standard solutions (Sigma Aldrich) (0–120 mg/L). All measurements were replicated three times.

Specifically, we used DPPH assay for measured antioxidant capacity of the lyophilized ethanolic and aqueous extracts subsequently obtained. Briefly, free radical scavenging capacity was determined using the DPPH assay, which is based on the decrease in DPPH- radical absorbance in the presence of antioxidant compounds [38]. Dry and ground samples (100 mg) were extracted with 70% (*v*/*v*) ethanol, and the extracts were mixed with a DPPH-in-methanol solution and kept in the dark for 30 min before reading at 517 nm. The antioxidant capacity was calculated by comparing the absorbance of the sample-DPPH mixtures with a control.

The ABTS assay was based on the decolorization of the ABTS-+ radical cation in the presence of compounds capable of donating electrons or hydrogen [39]. The ABTS-+ radical was generated by reacting ABTS and potassium persulfate solutions for 12–16 h and then diluting them in methanol until an appropriate absorbance was obtained at 734 nm. Extracts were prepared from 50 mg of the sample extracted with 70% (*v*/*v*) ethanol. An aliquot of the supernatant was mixed with the ABTS+ solution and incubated in the dark for 120 min before spectrophotometric reading at 734 nm.

Ferric reducing capacity was assessed using the FRAP assay, which measures the reduction of the ferric complex TPTZ–Fe^3+^ to its ferrous form TPTZ–Fe^2+^, evidenced by an increase in absorbance at 593 nm [39]. The FRAP reagent was prepared by mixing an acetate buffer (pH 3.6), a TPTZ solution in HCl, and a FeCl_3_ solution, maintaining the mixture at 37 °C before analysis. 70% ethanolic extracts were obtained from 50 mg of the sample, and an aliquot of the supernatant was incubated with the FRAP reagent for 30 min in the dark, after which the absorbance at 593 nm was recorded.

### 2.5. Cell Culture and Treatment Conditions

GES-1 cells were donated by Dr. Dawid Kidane-Mulat (Austin, TX, USA). The culture conditions were as described in previous publications [40]. The GES-1 cell line corresponds to an immortalized human gastric epithelial cell line commonly used as an in vitro model of gastric mucosal physiology and oxidative stress-related injury [40]. Cells were maintained in high-glucose Dulbecco’s Modified Eagle Medium (DMEM) supplemented with 10% fetal bovine serum (FBS) (#SV30160.03, Hyclone-Cytiva, Pasching, Austria) for GES-1 cells. The cells were maintained with penicillin/streptomycin (1X) (#15140122, Thermo Fisher Scientific, Canada) at 37 °C in a humidified 5% CO_2_ incubator. Regarding treatment conditions, the lyophilized extracts obtained from the six macroalgae were reconstituted in sterile water for all cell assays. GES-1 cells were incubated in DMEM supplemented with 5% FBS (control) and exposed to various concentrations of aqueous and ethanolic macroalgal extracts (50, 100, and 200 µg/mL) for 24 h [23,40,41]. Subsequently, to induce oxidative stress, cells were treated with 100 µM H_2_O_2_ for 3 h at 37 °C, according to established protocols [40]. The reduction of FBS from 10% (used for routine maintenance) to 5% during experimental conditions was implemented to minimize excessive cell proliferation and mitigate serum-derived interference during both oxidative stress induction and treatment evaluation.

### 2.6. Cell Viability Assay

We assess cell viability utilizing the MTS reagent, as it described in previous publications [40,42]. GES-1 cells were seeded in a 96-well plate. Once GES-1 cells reached approximately 70% of confluence, the cells were treated with different concentrations of ethanolic and aqueous extracts of each macroalgae (50, 100, and 200 µg/mL) for 24 h, and subsequently exposed to 100 µM H_2_O_2_ for 3 h. After this treatment, 20 µL MTS/PMS was added to each well (the final concentration of MTS was 333 µg/mL and that of PMS was 25 µM) for 1 h at 37 °C. The absorbance was measured at 490 nm using a microplate reader (NOVOstar, BMG LabTech, Ortenberg, Germany). The viability was expressed as the percentage of reduced MTS; the absorbance of control cells represented 100% cell viability.

### 2.7. Cellular ROS Level by Time-Lapse in Confocal Microscopy

We assessed reactive oxygen species (ROS) levels using the fluorescent probe DCFH-DA (2′,7′-dichlorofluorescein diacetate) in time-lapse confocal microscopy assay according to previous work [40]. GES-1 cells were seeded in a glass-bottom cell culture dish (NEST, Labmed, Wuxi, China). Upon reaching approximately 70% confluence, GES-1 cells were incubated in DMEM supplemented with 5% FBS (control) or treated with 200 µg/mL of *Gracilaria chilensis* ethanolic extract in the same medium. Following a 24 h incubation, cells were loaded with 10 µM DCFH-DA for 20 min at 37 °C, washed thrice with 1X PBS, and maintained in 1X HBSS buffer. Intracellular ROS imaging was performed using a laser scanning confocal microscope (LSCM-800) equipped with a 63×/1.46 oil immersion Plan-Apochromat objective, utilizing excitation and emission wavelengths of 485 nm and 536 nm, respectively. Initially, the basal state of both control and extract-treated cells was recorded. Subsequently, 100 µM H_2_O_2_ was added to both groups, and fluorescence kinetics were recorded every 30 s for 7 continuous minutes to monitor probe activation. Confocal images were captured at a resolution of 1024 × 1024 pixels (202.83 µm^2^) and analyzed using ZEN-2.1 software to define regions of interest (ROIs) per cell. Probe expression was quantified as the integrated mean green fluorescence intensity (488 nm channel) per cell (*n* = 30–40 cells). For each experimental group, the average integrated intensity was determined according to the following equation:

Average Integrated Intensity = Σ(NPc × MIc)/*n*

where NPc = number of pixels per cell, MIc = mean intensity per cell, and *n* = number of cells.

### 2.8. Apoptotic Assay by Cleaved Caspase 3 and Confocal Analysis

GES-1 cells were seeded on coverslips in 24-well plates. Immunofluorescence analysis was performed on confluent GES-1 cells. The cells (control or treated with *G. chilensis* ethanolic extracts, H_2_O_2_, *G. chilensis*/H_2_O_2_, and Cisplatin) were fixed with 4% paraformaldehyde for 10 min and washed with PBS 1X. The cells were permeabilized with 0.2% Triton™ X-100 for 15 min and blocked with 2% BSA for 1 h at room temperature. Cells were incubated with primary antibodies anti-Cleaved caspase 3 (1:500, Cell Signaling) overnight at 4 °C and washed three times for 15 min each time with PBS 1X. This was followed by incubation with the secondary antibody Alexa Fluor 488 (1:500, Thermo Fisher Scientific) for 1 h at room temperature, then labeling with DAPI for 10 min. Cells were washed three times with PBS 1X, and the coverslips were mounted on microscope slides using Fluoromount. The samples were examined with laser scanning confocal microscopy (LSCM-800) using 405 and 488 nm lasers and Plan Apochromat 63×/1.46 oil immersion objective. The confocal images were obtained with a size of 1024 × 1024 pixels (202.83 µm^2^). We selected a random sample of 10 cells per 3–4 images (*n* = 30–40 cells). We analyzed cleaved caspase-3 expression by measuring the integrated green fluorescence intensity (channel 488) per cell. The mean of the integrated intensity was calculated using the following formula:

Average Integrated Intensity = Σ(NPc × Mean intensity)/*n*, where NPc = number of pixels per cell, MIc = mean intensity per cell, and *n* = number of cells. The integrated intensity for each treatment was normalized with respect to the control group.

### 2.9. Statistical Analysis

All experiments were performed in triplicate, and data are expressed as mean ± S.E.M. Statistical analyses were performed using GraphPad Prism 8 software. Data were compared using a Student’s *t*-test for two-group comparisons, and a one-way ANOVA followed by Tukey’s post hoc test for multiple comparisons. Statistical significance was defined as *p* < 0.05 (*), *p* < 0.01 (**), *p* < 0.001 (***), and *p* < 0.0001 (****).

## 3. Results

### 3.1. Macroalgae Extracts Exhibit Antioxidant Capacity with Significant Polyphenol Levels

We collected six macroalgae species (green, brown, and red) along the Chilean coast during summer. These six seaweed raw samples were dried and pulverized for the antioxidant capacity assay, which were characterized by FRAP, ABTS, and DPPH assays (Table 2). All seaweeds exhibited significantly higher ABTS values compared with DPPH or FRAP. Moreover, we analyzed antioxidant capacity in aqueous and ethanolic extracts from the same six macroalgae species collected. This assay was assessed only with the DPPH method (Table 3). However, no significant differences in antioxidant capacity were observed between aqueous and ethanolic extracts from each macroalgae, except for *L. spicata*. These results suggest the presence of hydrophilic and lipophilic/organic antioxidants—such as polyphenols—that operate through mixed-mode reaction mechanisms [43,44]. Based on these results, the analysis of phenolic and flavonoid content in aqueous and ethanolic extracts (Table 4 and Table 5) shows that brown macroalgae *M. pyrifera* and red macroalgae *G. chilensis* have significantly higher phenolic levels compared with the other species (Table 4). Furthermore, the aqueous and ethanolic extracts of *G. chilensis* exhibited significantly higher flavonoid levels (1.70 and 2.20 mgEq Quercetin/g, respectively) compared with corresponding extracts of all other species.

### 3.2. Red Macroalgae Extract Protects the Cell Viability of Epithelial Gastric Cells Exposed to Oxidative Stress

**Figure 1:**
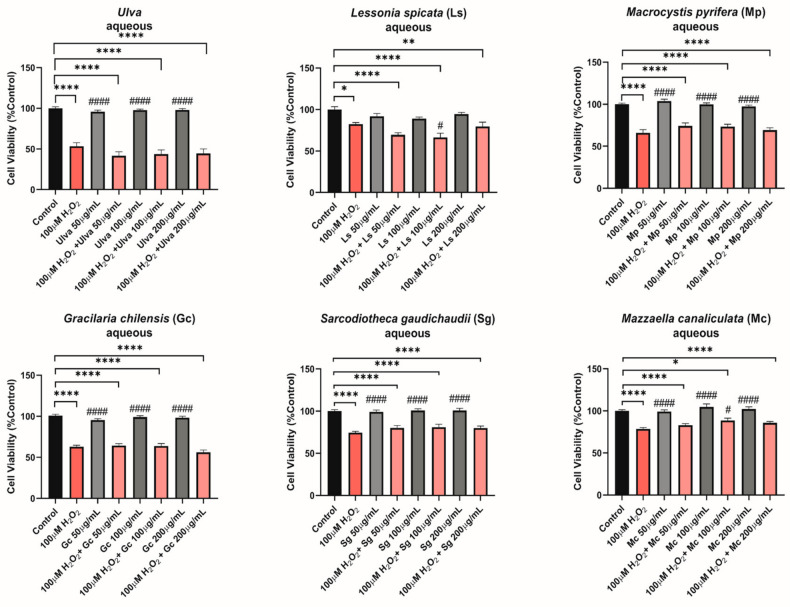
Effects of aqueous algae extracts on the viability of GES-1 cells exposed to hydrogen peroxide. Cell viability was assessed using the MTS assay in GES-1 cells. GES-1 cells were treated with different concentrations (50, 100, and 200 μg/mL) for 24 h of aqueous extracts of algae, such as *Ulva*, *Lessonia spicata*, *Macrocystis pyrifera*, *Gracilaria chilensis*, *Sarcodiotheca gaudichaudii*, and *Mazzaella canaliculata*, and exposed to 100 μM of hydrogen peroxide. Cell viability was expressed as a percentage of cell viability relative to the control. “*” represents a significant difference between each group and the control group, “#” represents a significant difference between each group and the peroxide group. Data are expressed as mean ± S.E.M., *n* = 3. * *p* < 0.05 vs. Control, ** *p* < 0.01 vs. Control, **** *p* < 0.0001 vs. Control, ^#^ *p* < 0.05 vs. H_2_O_2_, and ^####^ *p* < 0.0001 vs. H_2_O_2_.

**Figure 2:**
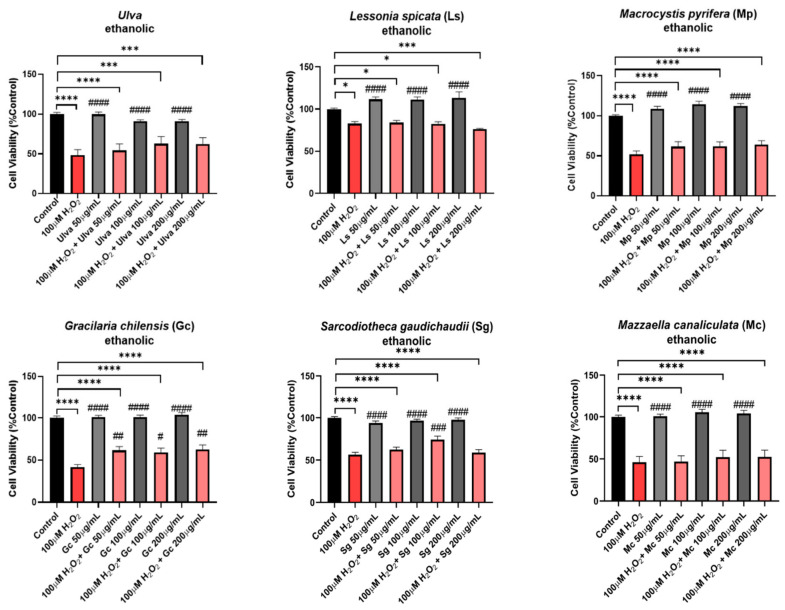
Effects of ethanolic algae extracts on the viability of GES-1 cells exposed to hydrogen peroxide. Cell viability was assessed using the MTS assay in GES-1 cells. GES-1 cells were treated with different concentrations (50, 100, and 200 μg/mL) for 24 h of ethanolic extracts of algae, such as *Ulva*, *Lessonia spicata*, *Macrocystis pyrifera*, *Gracilaria chilensis*, *Sarcodiotheca gaudichaudii*, and *Mazzaella canaliculata*, and exposed to 100 μM of hydrogen peroxide. Cell viability was expressed as a percentage of cell viability relative to the control. “*” represents a significant difference between each group and the control group, “#” represents a significant difference between each group and the peroxide group. Data are expressed as mean ± S.E.M., *n* = 3. * *p* < 0.05 vs. Control, *** *p* < 0.001 vs. Control, **** *p* < 0.0001 vs. Control, ^#^ *p* < 0.05 vs. H_2_O_2_, ^##^ *p* < 0.01 vs. H_2_O_2_, ^###^ *p* < 0.001 vs. H_2_O_2_ and ^####^ *p* < 0.0001 vs. H_2_O_2_.

To evaluate the protective effect of macroalgae extracts on gastric epithelial cell viability under oxidative stress, the GES-1 cell line was preincubated with aqueous and ethanolic extracts in the presence or absence of 100 μM hydrogen peroxide, following the method described in [40]. All aqueous and ethanolic extracts of the six macroalgae, at concentrations of 50, 100, and 200 μg/mL for 24 h, were non-toxic to GES-1 (Table 6). Notably, only red macroalgae extracts significantly protected GES-1 cell viability exposed to 100 μM hydrogen peroxide (Figure 1 and Figure 2). Specifically, the *M. canaliculata* aqueous extract, and the *G. chilensis* and *S. gaudichaudii* ethanolic extracts maintained cell viability during hydrogen peroxide exposure (Table 7). Preincubation with *M. canaliculata* increased GES-1 cell viability by 9.95% (*p* < 0.05), while *S. gaudichaudii* aqueous extracts led to a 17.78% increase in cell viability (*p* < 0.003) during peroxide exposure. Furthermore, *G. chilensis* ethanolic extracts, across all concentrations, increased GES-1 cell viability by 20.25% (*p* < 0.01), 17.99% (*p* < 0.05), and 21.33% (*p* < 0.01) during peroxide exposure. Consequently, preincubation of GES-1 cells with red macroalgae extracts for 24 h enhanced cell viability under oxidative stress.

### 3.3. Extract from Gracilaria Chilensis Reduces Cellular Oxidative Stress and Apoptosis Induced by Peroxide

**Figure 3:**
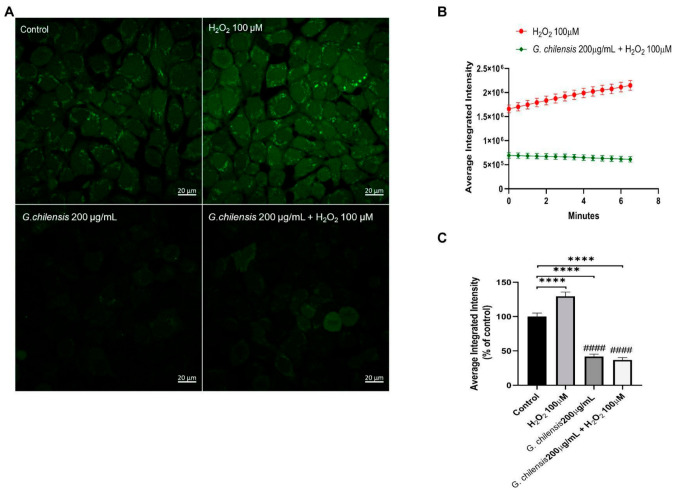
*Gracilaria chilensis* ethanolic extracts decrease intracellular ROS levels in GES-1 exposed to hydrogen peroxide. (**A**) The GES-1 cells were pretreated with 200 μg/mL *G. chilensis* ethanol extracts for 24 h and exposed to 100 μM of hydrogen peroxide for 3 h. The ROS levels were visualized using the DCFH-DA fluorescent probe, and images were acquired by confocal microscopy. Scale Bar: 20 μm. (**B**) The ROS levels were calculated by the integrated intensity of green fluorescence. The fluorescence intensity was expressed as the average integrated intensity per minute for each measurement (7 min). (**C**) The integrated intensity was expressed as a percentage relative to the control, with *n* = 20–40 cells per treatment. “*” represents a significant difference between each group and the control group, “#” represents a significant difference between each group and the peroxide group. Data are expressed as mean ± S.E.M., *n* = 20–40 cells. **** *p* < 0.0001 vs. Control, and ^####^ *p* < 0.0001 vs. H_2_O_2_.

Given that ethanolic extracts of *G. chilensis* provided superior protection of GES-1 cell viability against hydrogen peroxide, we evaluated whether these extracts could directly reduce ROS levels using the DCFH-DA fluorescent probe and time-lapse confocal microscopy (Figure 3). GES-1 cells preincubated with 200 μg/mL of *G. chilensis* ethanolic extracts for 24 h showed a decrease in ROS levels after incubation with 100 μM hydrogen peroxide over a 7 min time-lapse period (Figure 3B). Furthermore, preincubated GES-1 cells exhibited significantly reduced intracellular ROS levels (42%, *p* < 0.0001) compared to control cells (Figure 3C). A similar trend was observed in GES-1 cells preincubated with *S. gaudichaudii* ethanolic extracts (Supplementary Figure S1). Collectively, these findings demonstrate that preincubation with red macroalgae extracts, such as those from *G. chilensis,* decreases intracellular ROS levels, suggesting a protective mechanism against oxidative stress.

**Figure 4:**
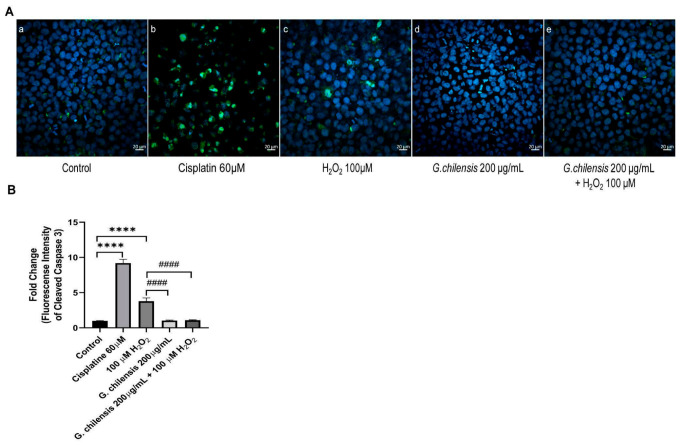
*Gracilaria chilensis* ethanolic extract protects against apoptosis in GES-1 cells exposed to hydrogen peroxide. (**A**) The cells were fixed and immunolabeled with anti-Cleaved Caspase 3 (Green) and DAPI (Blue). (**a**) Control cells without algae extracts; (**b**) Cells treated with 60 μM Cisplatine for 16 h (positive control); (**c**) Cells treated with 100 μM hydrogen peroxide for 3 h; (**d**) Cells treated with 200 μg/mL *G. chilensis* ethanolic extracts for 24 h; (**e**) Cells treated with 200 μg/mL *G. chilensis* ethanolic extracts for 24 h and exposed for 3 h to 100 μM hydrogen peroxide. Images were viewed by confocal microscopy. Scale bar: 20 μm. (**B**) Cleaved Caspase-3 expression was quantified by integrated green fluorescence intensity and was expressed as fold change with respect to the control group. “*” represents a significant difference between each group and the control group, “#” represents a significant difference between each group and the peroxide group. Data are expressed as mean ± S.E.M., *n* = 30–40 cells. **** *p* < 0.0001 vs. Control, and ^####^ *p* < 0.0001 vs. H_2_O_2_.

In light of the observed reduction in intracellular ROS levels, we further investigated whether this antioxidant effect correlates with a decrease in programmed cell death, as oxidative stress has been shown to induce apoptosis [45]. We evaluated apoptosis by assessing the cleaved caspase-3 levels in GES-1 cells preincubated with the macroalgae extracts and subsequently exposed, or not, to hydrogen peroxide (Figure 4). The *G. chilensis* ethanolic extracts showed a significantly reduced apoptosis (*p* < 0.0001) as indicated by active caspase 3 immunolabeling (Figure 4B), compared to positive controls with 60 μM cisplatin or 100 μM hydrogen peroxide.

## 4. Discussion

Oxidative stress is a recognized pathogenic driver of gastric diseases, including gastritis, ulcer development, and gastric cancer [46]. While current pharmacological treatments primarily target acid secretion or *Helicobacter pylori* eradication, they do not directly address oxidative injury to the gastric mucosa [42]. In this context, marine macroalgae-derived bioactive compounds, particularly when developed as nutraceuticals [8], represent a valuable complementary approach. Our findings address this gap by demonstrating the antioxidant and cytoprotective potential of Chilean macroalgae in a gastric epithelial cell model of oxidative stress. Red macroalgal crude extracts, through combined cytoprotective, ROS-reducing, and anti-apoptotic effects, emerge as promising nutraceutical candidates for the prevention of oxidative stress-related gastric diseases.

In the present study, we defined two criteria to identify macroalgae crude extracts with potential for preventing oxidative stress-related gastric diseases: in vitro antioxidant capacity (cell-free assays) and the antioxidant activity within the gastric epithelial cell line GES-1 exposed to hydrogen peroxide. We demonstrated that (i) Both aqueous and ethanolic extracts from macroalgae collected along the Chilean coast exhibit antioxidant properties in chemical assays (Table 2, Table 3 and Table 4), (ii) extracts obtained from red macroalgae, in contrast to green and brown species, provided significantly greater protection of GES-1 cell viability under oxidative stress (Figure 1 and Figure 2, and Table 7), and (iii) extracts from *G. chilensis* significantly attenuated apoptosis induced by oxidative stress, likely by reducing intracellular reactive oxygen species (ROS) (Figure 3 and Figure 4). Together, these results provide comparative evidence of the superior cytoprotective capacity of red macroalgae as a promising natural source of bioactive compounds for the prevention of gastric diseases associated with oxidative damage.

Our results showed that all macroalgae extracts exhibited antioxidant activity. In general, crude extracts exhibited antioxidant properties consistent with previous reports of similar macroalgae species from other regions [37,47,48,49,50,51,52]. Consequently, all macroalgae extracts contain compounds with antioxidant activity. Several antioxidant compounds have been identified in macroalgae, including sulfated polysaccharides (e.g., carrageenan, porphyran), carotenoids (lutein, zeaxanthin), lectins, phycobiliproteins, and polyphenols [49,53,54,55,56,57,58,59]. These substances may help preserve gastric mucosal integrity and maintain homeostasis by scavenging ROS, chelating metal ions, and inhibiting lipid peroxidation [22,26,56]. In this study, we focused on polyphenols (phenols and flavonoids) due to their known gastroprotective effects and their role in reducing gastric cancer risk [26,60]. Although both *M. pyrifera* and *G. chilensis* exhibit elevated phenolic content, only red macroalgae consistently exerted cytoprotective effects, indicating that chemical antioxidant capacity alone does not fully predict biological efficacy.

Preincubation with extracts from *G. chilensis*, *S. gaudichaudii*, and *M. canaliculata,* significantly preserved GES-1 cell viability under oxidative stress. The protective effect of *G. chilensis* ethanolic extracts correlates with their higher levels of phenolic and flavonoid content (Table 3 and Table 4), which could have a potential role in the prevention of different human diseases [61,62]. Although polyphenols often exhibit limited systemic bioavailability, they may reach high local concentrations in the gastrointestinal tract after oral administration, exerting protective effects on the gastric epithelium [60,63]. In addition, *G. Chilensis* ethanolic extracts reduced intracellular ROS levels and apoptosis in GES-1 cells, supporting their cytoprotective role. These results are consistent with previous in vivo studies on *Gracilaria* species [23,25,64,65] and extend them by providing comparative evidence across macroalgal groups under standardized conditions. The observed effects are likely not solely attributable to polyphenols alone. Other bioactive compounds, such as sulfated polysaccharides, carotenoids, and phycobiliproteins, may act synergistically with polyphenols to regulate redox homeostasis [53,66]. Future studies should focus on the isolation and characterization of these compounds and their potential synergistic interactions.

This study demonstrates the superior cytoprotective and antioxidant effects of red macroalgal extracts, although some limitations should be noted. The analysis was limited to in vitro assays using a single gastric epithelial cell line. Nevertheless, this work provides one of the first comparative evaluations of Chilean macroalgae in a gastric epithelial model of oxidative stress. Further studies are needed to characterize the active compounds and validate their efficacy in vivo. In addition, bioavailability, safety, and stability must be assessed to support their development as nutraceuticals. Future studies will also explore the mechanism by which ROS induced apoptotic pathways in these cell models. Overall, red macroalgae represents a promising source of bioactive compounds for the prevention of oxidative stress-induced gastric diseases and for diet-based preventive strategies.

## 5. Conclusions

Red macroalgal extracts showed superior antioxidant and cytoprotective effects compared to green and brown macroalgal extracts, as evidenced by enhanced cell viability, reduced ROS accumulation, and attenuated apoptosis in the gastric epithelial cell line.

The observed bioactivity suggests that red macroalgae contain unique metabolites, including polyphenols, which modulate ROS levels and apoptotic signaling pathways.

These findings support the potential of red macroalgal extracts as promising candidates for the development of nutraceuticals or preventive therapies targeting oxidative stress-related gastric disorders.

## Figures and Tables

**Table 1:** Macroalgae species, site, and date of collection.

Species	Distribution	Collection
*Ulva* (Green Macroalgae)	La Herradura Beach – 29° 59° 00.57″ S – 71° 21′ 55.67″ O	11 January 2023
*Lessonia spicata* (Brown Macroalgae)	Punta de Talca 30° 55′ 43.33″ S – 71° 40′ 18.74″ O	29 November 2022
*Macrocystis pyrifera* (Brown Macroalgae)	Punta de Talca 30° 55′ 43.33″ S 71° 40′ 18.74″ O	16 November 2022
*Gracilaria chilensis* (Red Macroalgae)	Changa Beach 29° 57′ 33.87″ S – 71° 19′ 57.15″ O	18 January 2023
*Sarcodiotheca gaudichaudii* (Red Macroalgae)	Changa Beach 29° 57′ 33.87″ S – 71° 19′ 57.15″ O	18 January 2023
*Mazzaella canaliculata* (Red Macroalgae)	El Pulpito, Caldera 27° 01′ 26.40″ S – 70° 48′ 12.08″ O	10 January 2023

**Table 2:** Average Antioxidant capacity of raw macroalgae measured by FRAP, ABTS, and DPPH methods.

Species	TEAC (µmol/g)		
	FRAP	ABTS	DPPH
*Ulva* (Green Macroalgae)	6.39 ± 0.07 ***	10.68 ± 0.16 ***	2.97 ± 1.12 ***
*Lessonia spicata* (Brown Macroalgae)	1.28 ± 0.05 ***	12.39 ± 0.49 ***	2.34 ± 0.55 *
*Macrocystis pyrifera* (Brown Macroalgae)	2.76 ± 0.04 ***	9.16 ± 0.15 ***	1.87 ± 0.46 ***
*Gracilaria chilensis* (Red Macroalgae)	1.61± 0.03 ***	6.59 ± 0.11 ***	3.18 ± 0.30 *
*Sarcodiotheca gaudichaudii* (Red Macroalgae)	4.59 ± 0.20 ***	8.91 ± 0.07 ***	5.10 ± 1.04 ^ns^
*Mazzaella canaliculata* (Red Macroalgae)	6.91 ± 0.02 ***	9.02 ± 0.07 ***	3.76 ± 0.91 ^ns^

Mean ± SD, *n* = 3. * *p* < 0.05 DPPH Method vs. FRAP Method, *** *p* < 0.001 FRAP Method vs. ABTS Method, *** *p* < 0.001 ABTS Method vs. DPPH Method, *** *p* < 0.001 DPPH Method vs. FRAP, and ns, not significant DPPH Method vs. FRAP Method.

**Table 3:** Antioxidant activity of aqueous and ethanolic macroalgae extracts by DPPH assay.

Species	Aqueous Extracts	Ethanolic Extracts
	TEAC (µmol/g)	TEAC (µmol/g)
*Ulva* (Green Macroalgae)	3.26 ± 0.72 ^ns^	2.97 ± 1.12 ^ns^
*Lessonia spicata* (Brown Macroalgae)	4.21 ± 0.52 ^ns^	5.10 ± 1.04 *^,^**
*Macrocystis pyrifera* (Brown Macroalgae)	2.54 ± 0.56 ^ns^	3.76 ± 0.91 ^ns^
*Gracilaria chilensis* (Red Macroalgae)	2.29 ± 0.64 ^ns^	3.18 ± 0.30 ^ns^
*Sarcodiotheca gaudichaudii* (Red Macroalgae)	2.21 ± 1.13 ^ns^	1.89 ± 0.46 ^ns^
*Mazzaella canaliculata* (Red Macroalgae)	2.33 ± 0.95 ^ns^	2.34 ± 0.55 ^ns^

Mean ± SD, *n* = 3. * *p* < 0.05 *L. spicata*_EtOH_ vs. *M. pyrifera* _AQ_, ** *p* < 0.01 *L. spicata*_EtOH_ vs. *M. canaliculata*_AQ_, ** *p* < 0.01 *L. spicata*_EtOH_ vs. *S. gaudichaudii*, and ** *p* < 0.01 *L. spicata*_EtOH_ vs. *G. chilensis* _AQ_. ns, not significant.

**Table 4:** Phenolic levels in aqueous and ethanolic macroalga extracts.

Species	Aqueous Extracts	Ethanolic Extracts
	mg eq. Gallic Acid dw	mg eq. Gallic Acid dw
*Ulva* (Green Macroalgae)	5.61 ± 0.23	5.34 ± 0.27
*Lessonia spicata* (Brown Macroalgae)	2.59 ± 0.05	2.79 ± 0.29
*Macrocystis pyrifera* (Brown Macroalgae)	14.85 ± 0.61 ***	11.74 ± 0.49 ***
*Gracilaria chilensis* (Red Macroalgae)	5.98 ± 0.28 ***	3.99 ± 0.50 ***
*Sarcodiotheca gaudichaudii* (Red Macroalgae)	2.41 ± 0.01	3.01 ± 0.10
*Mazzaella canaliculata* (Red Macroalgae)	2.01 ± 0.05	1.85 ± 0.10

Mean ± SD, *n* = 3. *** *p* < 0.001 *M. pyrifera*_AQ_ vs. *M. pyrifera*_EtOH_, *** *p* < 0.001 *M. pyrifera*_AQ and EtOH_ vs. All other Extracts _AQ and EtOH_, *** *p* < 0.001 *G. chilensis*_AQ_ vs. *G. chilensis*_EtOH_, and *** *p* < 0.001 *G. chilensis* _AQ and EtOH_ vs. All other Extracts _AQ and EtOH_.

**Table 5:** Flavonoid levels in aqueous and ethanolic macroalgae extracts.

Species	Aqueous Extracts	Ethanolic Extracts
	mg eq. Quercitin/g dw	mg eq. Quercitin/g dw
*Ulva* (Green Macroalgae)	1.23 ± 0.17	0.59 ± 0.22
*Lessonia spicata* (Brown Macroalgae)	0.66 ± 0.05	1.30 ± 0.06
*Macrocystis pyrifera* (Brown Macroalgae)	0.60 ± 0.09	1.36 ± 0.17
*Gracilaria chilensis* (Red Macroalgae)	1.78 ± 0.19 ***	2.24 ± 0.63 ***
*Sarcodiotheca gaudichaudii* (Red Macroalgae)	0.61 ± 0.08	0.59 ± 0.06
*Mazzaella canaliculata* (Red Macroalgae)	1.21 ± 0.26	1.66 ± 0.09

Mean ± SD, *n* = 3. *** *p* < 0.001 *G. chilensis* _AQ_ vs. All other Extracts _AQ_, and *** *p* < 0.001 *G. chilensis*_EtOH_ vs. All other Extracts _EtOH_.

**Table 6:** Summary of GES-1 cell viability following exposure to aqueous or ethanolic macroalgae extract for 24 h.

Species	% Protection (Aqueous)			% Protection (Ethanolic)		
	50 µg/mL	100 µg/mL	200 µg/mL	50 µg/mL	100 µg/mL	200 µg/mL
*Ulva* (Green Macroalgae)	−4.24 ^ns^	−2.18 ^ns^	−2.05 ^ns^	−0.32 ^ns^	−9.35 ^ns^	−9.22 ^ns^
*Lessonia spicata* (Brown Macroalgae)	−8.15 ^ns^	−10.99 ^ns^	−5.41 ^ns^	10.77 ^ns^	10.33 ^ns^	12.61 ^ns^
*Macrocystis pyrifera* (Brown Macroalgae)	3.76 ^ns^	0.00 ^ns^	−2.58 ^ns^	8.52 ^ns^	14.47 ^ns^	11.99 ^ns^
*Gracilaria chilensis* (Red Macroalgae)	−5.37 ^ns^	−1.82 ^ns^	−2.62 ^ns^	0.95 ^ns^	1.01 ^ns^	3.81 ^ns^
*Sarcodiotheca gaudichaudii* (Red Macroalgae)	−1.23 ^ns^	0.87 ^ns^	0.37 ^ns^	−6.13 ^ns^	−3.46 ^ns^	−2.25 ^ns^
*Mazzaella canaliculata* (Red Macroalgae)	−0.81 ^ns^	4.68 ^ns^	2.20 ^ns^	0.65 ^ns^	5.45 ^ns^	4.00 ^ns^

Mean ± S.E.M, *n* = 3. ns, not significant.

**Table 7:** Summary of GES-1 cell viability following 24 h preincubation of aqueous or ethanolic macroalgae extract and exposure to 100 μM hydrogen peroxide.

Species	% Protection (Aqueous)			% Protection (Ethanolic)		
	50 µg/mL	100 µg/mL	200 µg/mL	50 µg/mL	100 µg/mL	200 µg/mL
*Ulva* (Green Macroalgae)	−11.60 ^ns^	−9.63 ^ns^	−8.80 ^ns^	6.00 ^ns^	14.40 ^ns^	14.32 ^ns^
*Lessonia spicata* (Brown Macroalgae)	−12.87 ^ns^	−16.00 *	−2.85 ^ns^	0.68 ^ns^	−0.92 ^ns^	−7.28 ^ns^
*Macrocystis pyrifera* (Brown Macroalgae)	8.40 ^ns^	7.53 ^ns^	3.43 ^ns^	9.90 ^ns^	10.32 ^ns^	12.33 ^ns^
*Gracilaria chilensis* (Red Macroalgae)	1.40 ^ns^	0.86 ^ns^	−6.80 ^ns^	20.25 **	17.99 *	22.33 **
*Sarcodiotheca gaudichaudii* (Red Macroalgae)	5.53 ^ns^	6.49 ^ns^	5.43 ^ns^	5.89 ^ns^	17.78 ***	2.50 ^ns^
*Mazzaella canaliculata* (Red Macroalgae)	4.44 ^ns^	9.95 *	7.16 *	0.73 ^ns^	6.20 ^ns^	6.43 ^ns^

Mean ± S.E.M, *n* = 3. * *p* < 0.05 H_2_O_2_ vs. Extract + H_2_O_2_, ** *p* < 0.01 H_2_O_2_ vs. Extract + H_2_O_2_, *** *p* < 0.001 H_2_O_2_ vs. Extract + H_2_O_2_, ns, not significant.

## Data Availability

The original contributions presented in this study are included in the article/Supplementary Materials. Further inquiries can be directed to the corresponding author.

## Supplementary Materials

The following supporting information can be downloaded at: https://www.mdpi.com/article/10.3390/nu18121878/s1, Figure S1: *Sarcodiotheca gaudichaudii* ethanol extracts decreased the intracellular ROS levels of GES-1 exposed to H_2_O_2_; Figure S2: *Mazzaella canaliculata* ethanol extracts decreased the intracellular ROS levels of GES-1 exposed to H_2_O_2_.
