# Supplementary material for: Antioxidant and Cytoprotective Effects of Chilean Macroalgae Against Oxidative Stress-Induced Damage in Gastric Epithelial Cells
Source: Nutrients. 2026 Jun 10;18(12):1878. doi: 10.3390/nu18121878 (PMC13304658; doi:10.3390/nu18121878)
Supplement: Supplementary file 1 [file nutrients-18-01878-s001.zip › nutrients-4301810-supplementary.pdf]

Supplementary figures

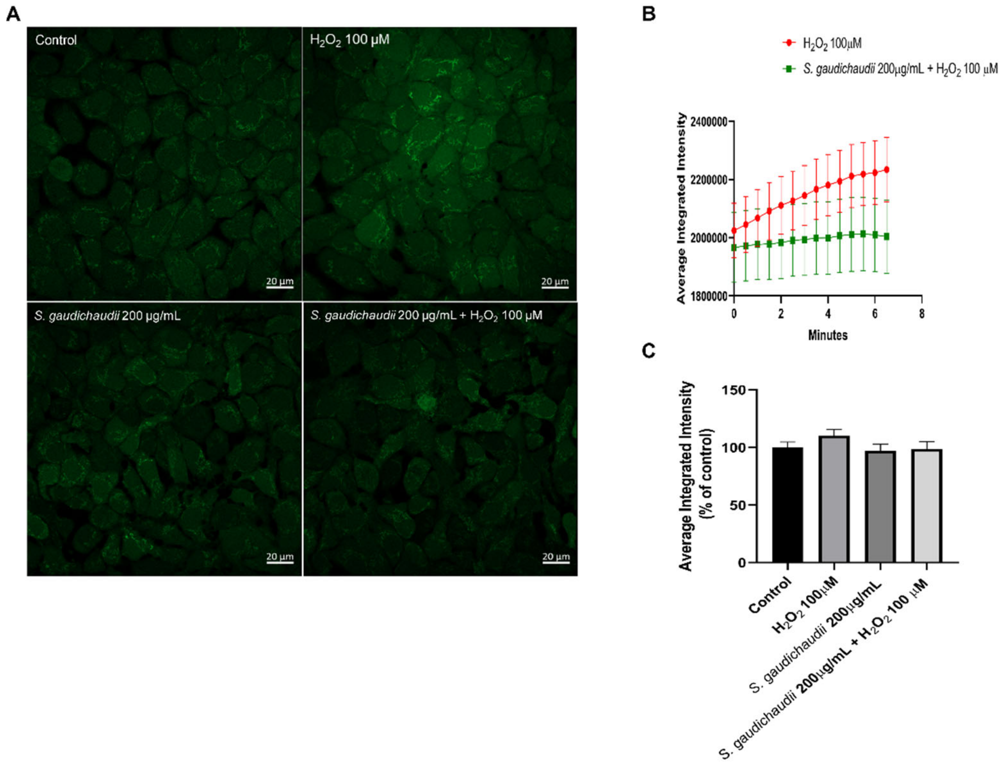

Figure S1. ***Sarcodiotheca gaudichaudii*** **ethanol extracts decreased the intracellular ROS levels of GES-1 exposed to H_2O_2. A)** The GES-1 cells were pretreated with 200 µg/mL *S. Gaudichaudii* ethanol extracts for 24 h and exposed to 100 µM of hydrogen peroxide for 3 h. The ROS levels were visualized using the DCFH-DA fluorescent probe, and images were acquired by confocal microscopy. Scale Bar: 20µm. **B)** The ROS levels were calculated by the integrated intensity of green fluorescence. The fluorescence intensity was expressed as the average integrated intensity per minute for each measurement (7 Minutes). **C)** The integrated intensity was expressed as a percentage relative to the control, with n= 20-40 cells per treatment.

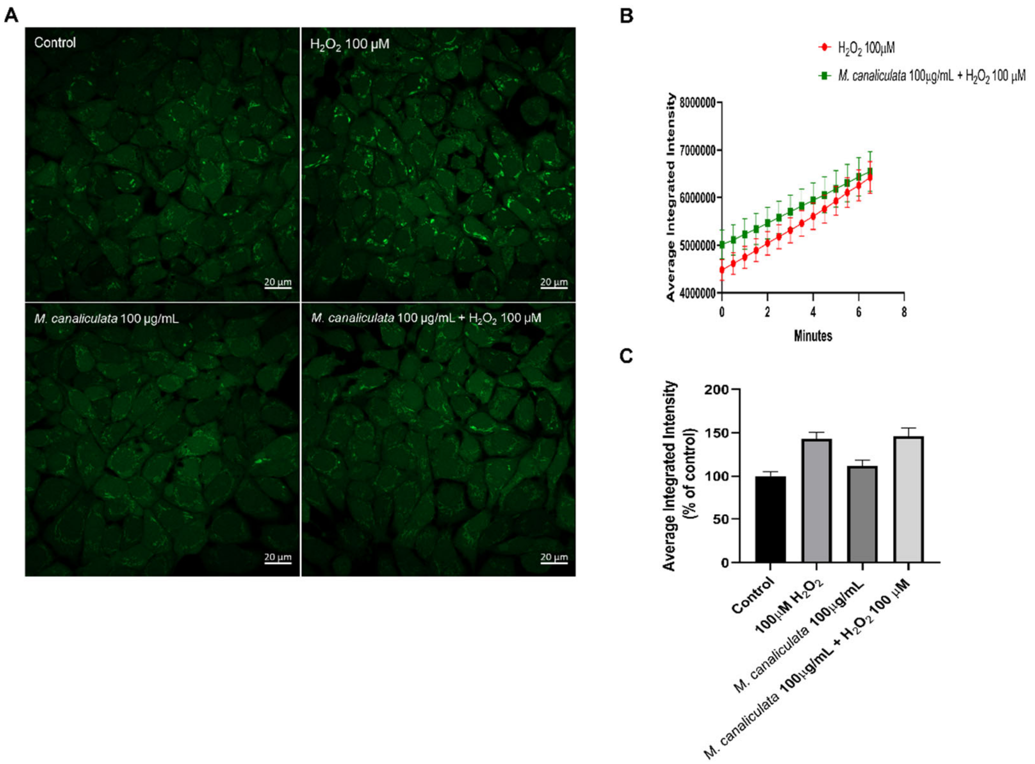

Figure S2. *Mazzaella canaliculata* ethanol extracts decreased the intracellular ROS levels of GES-1 exposed to H_2O_2. A) The GES-1 cells were pretreated with 200 µg/mL *M. canaliculata* ethanol extracts for 24 h and exposed to 100 µM of hydrogen peroxide for 3 h. The ROS levels were visualized using the DCFH-DA fluorescent probe, and images were acquired by confocal microscopy. Scale Bar: 20µm. **B)** The ROS levels were calculated by the integrated intensity of green fluorescence. The fluorescence intensity was expressed as the average integrated intensity per minute for each measurement (7 Minutes). **C)** The integrated intensity was expressed as a percentage relative to the control, with n= 20-40 cells per treatment.
